# Sprained Ankle

**Published:** 1909-06-19

**Authors:** 


### Orthopædics.

### SPRAINED ANKLE.

An ordinary " sprained ankle " is usually regarded as a very simple lesion—one to be dismissed with a short rest and the elements of palliative treatment. While no doubt it is elementary enough and generally yields to the few measures that are taken to treat it, there are occasions when it is advisable to pay a greater amount of attention to this lesion than it often receives. In itself and when properly attended to, it is not a very serious condition, but there are several points about its diagnosis and treatment which the practitioner would do well to bear in mind when called upon to handle a case.

In the first place, what is a sprained ankle? There is a degree of vagueness and looseness about the term which is to be regretted, since a variety of conditions which agree only in this that they are painful lesions in the neighbourhood of the ankle joint caused by strain or some kind of injury are included under it. Technically, where the term sprain is used, it is indicative of a simple stretching of tissues, ligamentous, muscular or bony, not amounting to actual rupture. Of late years, however, it has been found that in many of these so-called strains there existed actual solution of continuity in the tissue, in the shape of a partial rupture of muscle fibre, displacement of a tendon or ligament, or dislocation or subluxation of a joint. The juxta-epiphysial sprain described by Ollier has been found to be a much more serious lesion than it was held to be, owing to the fact that there is nearly always in such cases a fissure or fracture involving the juxta-epiphysial junction. Generally stated, sprains in children are more serious than in adults: a much lesser degree of violence is able to produce rupture of or damage to the child's tissues than is the case in the adult, and although the reaction that ensues upon such injuries may not be so great in the former as in the latter case, the ultimate results are apt to be worse where growing tissues are concerned. Sprains and strains of the foot are particularly difficult to classify or adequately to differentiate owing to the impossibility which often exists of definitely diagnosing the exact lesion. With the help of the $x$-rays a " sprained foot " can often be shown to be a relatively more important condition, showing a definite lesion of one of the metatarsals or of one or other of the tarsal bones; but without the invaluable aid which this means of ascertaining the condition of the joints and bones gives us, it is often extremely difficult to satisfy oneself that no more serious lesion than that usually included under the term sprain exists. Such indefinite conditions can all be classed as sprains, though very probably in the majority some definite rupture of tissue or fracture of bone is at the root of the trouble.

The practitioner is then first of all called upon to diagnose the case as one of sprained ankle—a matter which is not always easy. The typical signs and symptoms of such a lesion are sufficiently described in text-books, and need not be enumerated here. It is worth while, however, to point out that swelling and œdema are by no means uncommon after what are called simple sprains in the region of the ankle. Such œdema and swelling may extend over the whole foot, making it exceedingly difficult to find the ordinary bony landmarks and for the examiner to satisfy himself as to their integrity. In cases where the history of injury is wanting or slight, a sprain may be confounded with a much more serious condition such as tubercular disease, acute rheumatism starting in the ankle, or some form of acute arthritis or tenosynovitis. Again, the converse holds good, and a grave lesion may be regarded as a simple sprain and treated as such with disastrous consequences. This is especially the case when the patient states, as he so very often does, that the condition is due to an injury. Every sick person attempts to find a cause for his complaint, and orthopædic lesions are no exception to this rule. Thus a patient may come to the surgery with the self-made diagnosis of sprained ankle and a history of a side-slip a couple of days previously, and on examination the case may turn out to be one of tubercular disease. This point is merely referred to here to show the necessity of carefully examining each case of alleged sprain. Every means that the practitioner has at his disposal should be used to exclude any more serious lesion before the diagnosis of a simple sprain is made. The $x$-rays render very useful service, but they are not always at hand, but where any doubt exists as to the possibility of a more serious lesion being present, its existence should be taken for granted and the case treated accordingly. Where direct force has occasioned the injury this is specially important, but in that case a diagnosis can usually be made with care. It is the sprain caused by a wrench or slip, in which the pain of the strained ligamentous tissues overshadows the other symptoms, that the exclusion of fracture or rupture is particularly difficult.

There are some aspects of a sprained ankle which should never be lost sight of. One of these is the possible complications which may result in the shape of tenosynovitis or, in old patients, osteo-arthritis. In many cases a comparatively simple sprain of the ankle, which in another person would have led to no serious result, gives rise to the most obstinate cases of metatarsalgia. The connection between injuries in the ankle region and these indistinct pains which are located in the mid-tarsal region is not very clear, but such results are not very uncommon. In tabetic patients, whose primary lesion may be quite unsuspected, the injury may of course lead to a neuropathic arthritis: so also in a patient with a " tubercular diathesis " the most common cause of a joint lesion appears to be a simple sprain. Serious sprains very often lead to a weakening of the arch of the foot and to a bad condition of flat foot, which later on demands careful attention. Where a wound exists sepsis may occur and induce serious consequences, but this is rare in cases which can be considered as having been primarily simple sprains. Later sequelæ, in the majority of cases due to bad treatment, are limitation of movement at the joint or immobility, and chronic local pain which sometimes proves very obstinate.

(*To be continued.*)

